# Distance to High-Voltage Power Lines and Risk of Childhood Leukemia – an Analysis of Confounding by and Interaction with Other Potential Risk Factors

**DOI:** 10.1371/journal.pone.0107096

**Published:** 2014-09-26

**Authors:** Camilla Pedersen, Elvira V. Bräuner, Naja H. Rod, Vanna Albieri, Claus E. Andersen, Kaare Ulbak, Ole Hertel, Christoffer Johansen, Joachim Schüz, Ole Raaschou-Nielsen

**Affiliations:** 1 Danish Cancer Society Research Center, Copenhagen Ø, Denmark; 2 Danish Building Research Institute, Aalborg University, Construction and Health, Copenhagen SV, Denmark; 3 Social Medicine Section, Department of Public Health, University of Copenhagen, Copenhagen K, Denmark; 4 Risø National Laboratory for Sustainable Energy, Radiation Research Division, Technical University of Denmark, Roskilde, Denmark; 5 National Institute of Radiation Protection, Herlev, Denmark; 6 Department of Environmental Science, Aarhus University, Roskilde, Denmark; 7 Department for Environmental, Social and Spatial Change (ENSPAC), Roskilde University, Roskilde, Denmark; 8 Oncology Clinic, Finsen Centre, Rigshospitalet 5073, University of Copenhagen, Copenhagen Ø, Denmark; 9 International Agency for Research on Cancer (IARC), Section of Environment and Radiation, Lyon, France; The Ohio State University, United States of America

## Abstract

We investigated whether there is an interaction between distance from residence at birth to nearest power line and domestic radon and traffic-related air pollution, respectively, in relation to childhood leukemia risk. Further, we investigated whether adjusting for potential confounders alters the association between distance to nearest power line and childhood leukemia. We included 1024 cases aged <15, diagnosed with leukemia during 1968–1991, from the Danish Cancer Registry and 2048 controls randomly selected from the Danish childhood population and individually matched by gender and year of birth. We used geographical information systems to determine the distance between residence at birth and the nearest 132–400 kV overhead power line. Concentrations of domestic radon and traffic-related air pollution (NO_x_ at the front door) were estimated using validated models. We found a statistically significant interaction between distance to nearest power line and domestic radon regarding risk of childhood leukemia (p = 0.01) when using the median radon level as cut-off point but not when using the 75^th^ percentile (p = 0.90). We found no evidence of an interaction between distance to nearest power line and traffic-related air pollution (p = 0.73). We found almost no change in the estimated association between distance to power line and risk of childhood leukemia when adjusting for socioeconomic status of the municipality, urbanization, maternal age, birth order, domestic radon and traffic-related air pollution. The statistically significant interaction between distance to nearest power line and domestic radon was based on few exposed cases and controls and sensitive to the choice of exposure categorization and might, therefore, be due to chance.

## Introduction

In 2001 a working group at the International Agency for Research on Cancer (IARC) classified exposure to extremely low-frequency magnetic fields (ELF-MF) as ‘possibly carcinogenic to humans’ and exposure to extremely low-frequency electric fields were grouped as ‘not classifiable as to its carcinogenicity to humans’ [1]. The classification of ELF-MF was primarily based on epidemiological findings, showing an association between residential exposure to ELF-MF and childhood leukemia [2]. There is no known biological explanation for this association and the epidemiological findings have not been supported by animal studies [3]. Therefore, it is not known whether the observed association reflects a causal relationship or is due to bias, confounding or chance [2], [3].

In 2005 a large-scale case-control study from Great Britain [4] showed an association between proximity of residence at birth to high-voltage power lines and the risk of childhood leukemia. The association extended beyond distances where the ‘power line’-induced ELF-MF exceed background levels, which suggests that the association was not explained by the magnetic field but perhaps by some other risk factor. Several studies have looked for potential confounders which could explain the observed association between ELF-MF and childhood leukemia [5]–[18] including socioeconomic status, residential mobility, residence type, viral contacts, environmental tobacco smoke, dietary agents, and traffic density; but none of them appear to explain the association [19]. Little is known about the etiology of childhood leukemia and there are only few established causes of childhood leukemia including exposure to ionizing radiation (X-rays and gamma rays) and certain genetic diseases such as Down’s syndrome [20], [21]. The limited knowledge of the etiology of childhood leukemia makes it difficult to exclude the possibility of some yet unknown risk factor or of the combination of a number of risk factors, which could confound the analysis between ELF-MF and childhood leukemia [19].

The lack of an accepted biological explanation for the observed association between ELF-MF and childhood leukemia have raised doubt about the causality of the association. Several mechanisms of how extremely low-frequency electric and magnetic fields can cause cancer have, however, been proposed [3]. One hypothesis is that the electric field from power lines interacts with airborne pollutant particles and thereby increases the harmful effect of these particles [22]–[25]. Airborne particles such as tobacco smoke, radon decay products, chemical pollutants, spores, bacteria and viruses might all affect health. These particles can be deposited on the skin or in the airways by inhalation [3], [25]. In 1999 Fews et al. [23] found a higher deposition of pollutant aerosols on the body under high-voltage power lines, which they argued was due to the oscillation of charged particles in the electric field from the power line. Further, Fews et al. [24] suggested that the electric field from high-voltage power lines can cause electrical breakdown of the air resulting in emission of clouds of positive and negative ions, which can charge particles that pass through them. Since charged particles are more likely than uncharged particles to be deposited when close to the walls of the respiratory airways or to the skin, this could increase the exposure and thereby the adverse health effect of such particles.

The major objective of our study was, therefore, to investigate possible interactions between distance from residence to nearest power line and domestic radon and traffic-related air pollution, respectively, in relation to childhood leukemia risk; to our knowledge this has not previously been investigated. Additionally, we investigated whether adjusting for potential confounding factors alters the association between distance from residence at birth to nearest power line and childhood leukemia. We adjusted for factors observed to be associated with childhood leukemia risk in other studies, specifically socioeconomic status of the municipality, urbanization, maternal age, birth order, domestic radon and traffic-related air pollution [20], [21], [26], [27], which might also be related to distance from residence to nearest power line. The main effect of distance to nearest power line and childhood leukemia risk has been reported in a previous paper [28].

## Materials and Methods

### Ethics statement

The Danish Data Protection Agency (2007-41-0239) approved the study. In accordance with Danish law written consent was not obtained as the study was entirely register-based and did not involve biological samples from, or contact with study participants.

### Cases and controls

We identified all children in Denmark diagnosed with leukemia (all types) before the age of 15 years during the period 1968–1991 (inclusively) through the virtually complete nationwide Danish Cancer Registry [29]. Children with a previous cancer diagnosis were excluded. Two controls for each case were individually matched by gender and year of birth and were selected by incidence density sampling from the Danish Civil Registration System. All children born in Denmark, who were alive, without cancer and living in Denmark at the time of diagnosis of their matched case were eligible to become a control in the study.

We identified 1024 cases diagnosed with leukemia and selected 2048 matched controls, constituting a population of 3072 children (Figure S1).

### Distance to nearest power line

The addresses of cases and controls at birth were obtained from the Danish Civil Registration System and we identified geographical coordinates by linkage to the Danish Address Database. The geographical coordinates refer to the front door of each residence (for apartments the main entrance door) and are precise within a few meters. We collected data on existing and historical 132–400 kV overhead power lines with alternating current from the seven Danish transmission companies. The mapped grid includes 4336 km of current and historical power lines (23.1% 400 kV, 0.9% 220 kV, 46.3% 150 kV, 29.8% 132 kV). In addition information on the date when the line was put into and out of operation was collected. For the lines for which we only had information on the year of operation (77.5%), we set the date of operation to 31 December and the date when the line was put out of operation was set to 1 January. This restrictive approach was applied to avoid assigning exposure to unexposed individuals. The distance from residence at birth to the nearest power line that existed at the date of birth was calculated in ArcGIS 9.3. We successfully calculated distance for 2797 (91.0%) of the addresses at birth. Distance could not be calculated due to missing information in the address for 100 (9.8%) cases and 175 (8.5%) controls (Figure S1). Distance was categorized into three groups: 0–199 meters, 200–599 meters and ≥600 meters, in accordance with the categorization used by Draper et al. [4].

### Domestic radon

Domestic radon concentration at address at birth was estimated using a validated regression model constructed to predict radon concentrations in Danish dwellings on the basis of register data including geographical region, soil type, and house characteristics such as type of house, floor, basement, and building materials. Details on the model are given in previous papers [30], [31].

Exposure to domestic radon at address at birth was successfully estimated for 2904 (94.5%) addresses. We could not estimate domestic radon exposure for 62 (6.1%) cases and 106 (5.2%) controls (Figure S1).

### Traffic-related air pollution

We used the sum of nitrogen oxide gasses, NO_x_ (nitrogen monoxide (NO) + nitrogen dioxide (NO_2_)), as an indicator for air pollution from traffic because NO_x_ correlates strongly with other traffic-related pollutants in Danish streets, especially ultrafine PM (Particulate matter): *r* = 0.93 for total particle number concentration (size, 10–700 nm) and *r* = 0.70 for PM_10_ (mass of particles with an aerodynamic diameter less than 10 µm) [32], [33]. The average concentration of NO_x_ at the front door of the address at birth was estimated by use of the Operational Street Pollution Model taking into account both the street-level pollution as well as the background pollution [34], [35], [36], [37]. In short the model was used to estimate the air pollution level from street information (such as width of the street, the height of the buildings, the distance between building and street sections with no buildings), the traffic density on the street, the proportion of vehicles weighing more than 3,500 kg, the average speed in combination with information on emission factors for the Danish car fleet, meteorological variables (wind speed, temperature and solar radiation) and background level of air pollution at the front door.

Exposure to traffic-related air pollution at the birth address was successfully estimated for 2942 (95.8%) addresses and could not be estimated for 45 (4.4%) cases and 85 (4.2%) controls (Figure S1).

### Potential confounders

Socioeconomic status of the municipality (the average gross income of the municipality) has been described in detail previously [27], where it was estimated for children born in the period 1976–1991. Children born in the period 1953–1975 were assigned the municipality income level based on data in 1976. Definition of rural and urban area was based on the official Danish classification. Information on maternal age and birth order was provided by linkage with the Danish Civil Registration System using the personal identification number. We also adjusted the analyses for domestic radon and traffic-related air pollution.

We had no information on socio-economic status of the municipality for 6 cases and 12 controls, on urbanization for 100 cases and 175 controls, on maternal age for 43 cases and 77 controls and on birth order for 43 cases and 78 controls (Figure S1).

### Statistical analysis

Because of the nested case-control design of the study, with equal follow-up time for a case and the matched controls, the rate ratios [38] (hereafter denoted relative risks (RR)) for childhood cancer and 95% confidence intervals (CIs) were estimated by conditional logistic regression models with the “PROC PHREG” procedure in SAS 9.2.

We analyzed the association between distance to nearest power line and risk of childhood leukemia both with and without adjustment for potential confounders. The potential confounders included socio-economic status of the municipality (categorical; <10 pct., 10–90 pct., >90 pct.), urbanization (categorical; countryside, urban area), maternal age at time of birth (categorical; <30y, ≥30y), birth order (categorical; 1, ≥2), domestic radon (categorical; <50 pct., 50–90 pct., >90 pct.) and traffic-related air pollution (categorical; <50 pct., 50–90 pct., >90 pct.).

We analyzed possible interactions between distance to power lines and domestic radon and traffic-related air pollution, respectively. Since there is no known biological relevant cut-off point for radon or NO_x_, we dichotomized domestic radon and traffic-related air pollution using the median value as cut-off; in sensitivity analyses we used the 75^th^ percentile as cut-off point. In an additional sensitivity analysis we divided radon and NO_x_ into tertiles in order to investigate a potential dose-response association. All interaction analyses were adjusted for the potential confounders: socioeconomic status of the municipality, urbanization, maternal age at time of birth, birth order and domestic radon and traffic-related air pollution, respectively. From the former study on radon [31] we had information on radon for cases and controls until 1994 and therefore we conducted an additional sensitivity analysis (unadjusted) of the interaction between power lines and radon to maximize the power. Furthermore, the interaction was analyzed including radon as a continuous variable. The p-value for the interaction was obtained by comparing and testing the model with the interaction term against the model with only the main effect by means of the likelihood ratio test.

Due to small samples in some of the categories in the analysis of the interaction between distance to power line and radon, exact methods for logistic regression were also applied using LogXact. It was only possible to compute models without adjustment for confounding factors.

## Results

We excluded 139 (13.6%) cases and 248 (12.1%) controls due to missing data on distance to power line or any of the potential confounding factors: domestic radon, traffic-related air pollution, socioeconomic status of the municipality, urbanization, maternal age or birth order. Further, we excluded 6 cases and 179 controls since they no longer had a matched control or case, leaving 879 (85.8%) cases and 1621 (79.2%) controls for the analyses (Figure S1).

In Table 1 the distribution of potential confounders by case-controls status and by distance to nearest power line is shown. Cases were more likely than controls to be living in a municipality with a lower socioeconomic status at time of birth, to have a mother at the age of 30 or older at time of birth, and to have at least one older sibling. Cases and controls were similar regarding exposure to domestic radon and traffic-related air pollution at address at birth and regarding whether they were living in a town or at the countryside at the time of birth. Children living close to power lines tended to live in the countryside, to be exposed to higher concentrations of domestic radon, and to be exposed to smaller concentrations of air pollution than children living further away. Children living close to power lines were similar to children living far away regarding socioeconomic status of the municipality, maternal age and birth order.

**Table 1:** Potential confounders by case-control status and distance to nearest power line.

	Case-control status				P-value(χ^2^-test)	Distance to nearest power line (meters)						P-value (χ^2^-test)
	Cases		Controls			0–199		200–599		≥600		
	N	(%)	N	(%)		N	(%)	N	(%)	N	(%)	
Socioeconomic status					0.02							0.15
10% most poor	46	(5.2)	52	(3.2)		0	(0.0)	1	(0.9)	97	(4.1)	
80% middle group	739	(84.1)	1362	(84.0)		19	(86.4)	86	(81.1)	1996	(84.2)	
10% most rich	94	(10.7)	207	(12.8)		3	(13.6)	19	(17.9)	279	(11.8)	
Urbanization					0.63							0.01
Town	639	(72.7)	1193	(73.6)		15	(68.2)	65	(61.3)	1752	(73.9)	
Country side	240	(27.3)	428	(26.4)		7	(31.8)	41	(38.7)	620	(26.1)	
Maternal age (years)					0.01							0.89
<30	644	(73.3)	1260	(77.7)		16	(72.7)	82	(77.4)	1806	(76.1)	
≥30	235	(26.7)	361	(22.3)		6	(27.3)	24	(22.6)	566	(23.9)	
Birth order					0.02							0.62
1	363	(41.3)	746	(46.0)		8	(36.4)	44	(41.5)	1057	(44.6)	
>1	516	(58.7)	875	(54.0)		14	(63.6)	62	(58.5)	1315	(55.4)	
Domestic radon (Bq/m^3^)^1^					0.77							<0.01
<42	432	(49.2)	818	(50.5)		7	(31.8)	26	(24.5)	1217	(51.3)	
42–101	360	(41.0)	640	(39.5)		13	(59.1)	62	(58.5)	925	(39.0)	
≥102	87	(9.9)	163	(10.1)		2	(9.1)	18	(17.0)	230	(9.7)	
NO_x_ at the front door (ppb)^1^					0.21							0.04
<9	460	(52.3)	790	(48.7)		13	(59.1)	62	(58.5)	1175	(49.5)	
9–20	338	(38.5)	662	(40.8)		8	(36.4)	42	(39.6)	950	(40.1)	
≥21	81	(9.2)	169	(10.4)		1	(4.6)	2	(1.9)	247	(10.4)	
Total	879	(100.0)	1621	(100.0)		22	(100.0)	106	(100.0)	2372	(100.0)	

1 Cut-point is the 50^th^ and 90^th^ percentile.

Table 2 presents the crude and adjusted associations between distance to power lines and childhood leukemia. Adjusting for potential confounders had virtually no effect on the estimated association between distance to nearest power line and childhood leukemia.

**Table 2:** Crude and adjusted RRs for leukemia in association with distance to nearest power line.

Distance tonearest powerline (meters)	Cases	Controls	Total	Model 1^a^		Model 2^b^		Model 3^c^		Model 4^d^	
	N (%)	N (%)		RR (CI)	P-value	RR (CI)	P-value	RR (CI)	P-value	RR (CI)	P-value
0–199	10 (1.1)	12 (0.7)	22	1.65 (0.71–3.83)		1.77 (0.76–4.11)		1.73 (0.74–4.02)		1.68 (0.72–3.92)	
200–599	29 (3.3)	77 (4.8)	106	0.68 (0.44–1.05)		0.69 (0.45–1.07)		0.70 (0.45–1.09)		0.70 (0.45–1.09)	
≥600	840 (95.6)	1532 (94.5)	2372	1.00	0.11	1.00	0.11	1.00	0.13	1.00	0.13
Total	879 (100.0)	1621 (100.0)	2500								

a The crude model.

b Adjusted for socioeconomic status and urbanization.

c Adjusted for the same as model 2 and for maternal age and birth order.

d Adjusted for the same as model 3 and for domestic radon and air pollution.

Table 3 shows the respective joint effects of distance to nearest power line and domestic radon and air pollution on leukemia. We found a statistically significant interaction between distance to nearest power line and domestic radon (p = 0.01). The RR for children living 0–199 meters of a power line and being exposed to domestic radon ≥42 Bq/m^3^ was 2.88 (95% CI: 1.01–8.27) compared to children living ≥600 meters from a power line and exposed to domestic radon <42 Bq/m^3^. Children living 200–599 meters from a power line and being exposed to domestic radon <42 Bq/m^3^ had a lower risk with an RR of 0.24 (95% CI: 0.07–0.83) compared to children living ≥600 meters from a power lines and exposed to domestic radon <42 Bq/m^3^. There was no evidence of interaction between distance to nearest power line and traffic-related air pollution (p = 0.73). The sensitivity analysis including domestic radon and traffic-related air pollution with a cut-off at the 75^th^ percentile showed no significant interaction with domestic radon (p = 0.90) or air pollution (p = 0.59) (Tables S1) and there was no dose-response association when radon and air pollution were divided into tertiles (Table S2). The interaction between power lines and radon remained statistically significant in the sensitivity analysis including radon until 1994, however, the RR for children exposed to radon concentrations of ≥42 Bq/m^3^ and living within 200 meters of a power line was no longer statistically significant (RR = 2.62 (95% CI: 0.97–7.07)) (Table S3). When radon was included as a continuous variable the interaction between distance to power line and radon was not statistically significant (p = 0.37) (data not shown). The result of the interaction analysis using exact methods was similar to the result of the unadjusted analysis using asymptotic methods (both presented in Table S4). The RR for children living within 200 m of a power line and exposed to radon concentrations of ≥42 Bq/m^3^ was still elevated but not statistically significant.

**Table 3:** The joint effects of distance to nearest power line and domestic radon and air pollution, respectively, on leukemia.

	Adjusted			
	RR (95% CI)	(N cases; N controls)		P-value for interaction
	Distance (meters)			
	0–199	200–599	≥600	
Domestic radon (Bq/m^3^)^1^^,^ 2				
<42	0.33 (0.04–2.80)	0.24 (0.07–0.83)	1.00	0.01
	(1; 6)	(3; 23)	(428; 789)	
≥42	2.88 (1.01–8.27)	0.85 (0.52–1.39)	0.95 (0.79–1.16)	
	(9; 6)	(26; 54)	(412; 743)	
NO_x_ at the front door (ppb)^1^^,^ 3				
<9	2.21 (0.73–6.64)	0.68 (0.38–1.21)	1.00	0.73
	(7; 6)	(17; 45)	(436; 739)	
≥9	1.02 (0.25–4.17)	0.67 (0.34–1.33)	0.92 (0.76–1.11)	
	(3; 6)	(12; 32)	(404; 793)	

1 Cut-point is the median.

2 The adjusted analysis includes following potential confounders: socioeconomic status, urbanization, maternal age, birth order and air pollution.

3 The adjusted analysis includes following potential confounders: socioeconomic status, urbanization, maternal age, birth order and domestic radon.

## Discussion

We found a statistically significant interaction between distance to nearest power line and domestic radon regarding risk of childhood leukemia when using the median radon level as cut-off point but not when using the 75^th^ percentile of radon exposure as cut-off point. We found no evidence of an interaction between distance to nearest power line and traffic-related air pollution. We found almost no change in the estimated association between distance to power lines and risk of childhood leukemia when adjusting for socioeconomic status of the municipality, urbanization, maternal age, birth order, domestic radon and traffic-related air pollution.

### Strengths and limitations

Our study was a register-based case-control study covering the entire population of Denmark. Cases were identified in a virtually complete national cancer registry, and the Central Population Registry provided an excellent basis for an unbiased sampling of controls. The potential for selection bias in our study is, therefore, minimal. We identified around 90% of current as well as historical power lines with ≥132 kV and according to two of the transmission companies (covering approximately 25% of all power lines) the data on power lines had a precision of 3–5 meters. The geographical coordinates used to identify the front door at the address at birth had a precision of few meters. For apartments the main entrance door was identified, which for the majority of apartments is within 5 meters, however, for some types of apartments a larger deviation may be expected.

Distance to nearest power line, domestic radon and air pollution was estimated for the address at birth, although exposure accumulated over all childhood addresses might be more relevant [31]. However, since interactions were in focus in the present study, we included the exposures at birth addresses only to ensure that the exposure to power lines (distance from residence) and exposure to domestic radon and traffic-related air pollution was present at the same address, which is essential for the analysis of interaction.

It is a limitation of the study that the analyses of interaction were based on few cases and controls for some of the cells of table 3. Data on air pollution was available from a previous study for children diagnosed during the period 1968–1991. It was not feasible to collect data for an extended period, though it would have contributed with more cases and controls and thereby more power in the analyses.

Exposure to domestic radon and traffic-related air pollution at the birth address were both modeled using successfully validated prediction models which have been applied in previous studies [31], [39]–[41]. Radon values predicted by the model have previously been validated against measured values: Some non-differential misclassification occurs, but 80% of the lowest exposures (<50 Bq/m^3^) were correctly classified and 60% of the highest exposures (>100 Bq/m^3^) were correctly classified [31]. The internationally widely used [36] Operational Street Pollution Model used to predict the NO_x_ concentration at the front door has been validated by comparing the calculated nitrogen dioxide (NO_2_) values with measured values at 204 locations in Denmark with an R^2^ value of 0.82 [42]. Even though more precise estimates for exposure may be obtained with measured values, modeled estimates make it possible to avoid participation bias and to obtain a larger amount of participants than would be possible with measurement in each house.

### Comparison with other studies and interpretation

In a previous study, where we included all children diagnosed with leukemia before the age of 15 years during the period 1968–2006, we report a OR of 0.76 (95% CI: 0.40–1.45) for children who lived 0–199 meters from the nearest power line compared with children who lived ≥600 meters away [28]. In the present study we report an RR of 1.68 (95% CI: 0.72–3.92) when living 0–199 meters from the nearest power line compared to those living ≥600 from the nearest power line. The 95% CIs of the two estimates widely overlap and none of the estimates are statistically significant, which indicate that the different estimates are due to chance and are two versions of the same null-result.

We found a statistically significant interaction between distance from residence to nearest power line and domestic radon but no interaction between distance and traffic-related air pollution. The interaction with radon showed a higher risk of childhood leukemia if living close to power lines and being exposed to high radon concentrations. This is in line with the hypothesis by Fews et al. [24] that ions of positive and negative charge emitted from power lines increases the proportion of charged radon particles and thereby increases the deposition of radon decay products on the skin or in the airways. Thus, the hypothesis by Fews et al. could explain the interaction observed in the present study. On the other hand the effect of the ions-clouds is expected to have the highest impact on particles arising outdoors whilst the exposure to radon is highest indoor where it can concentrate. According to an independent Advisory Group on Non-Ionising Radiation, advising The National Radiological Protection Board (NRPB) in UK, some of the ions are expected to enter the houses but the effect of the ion-clouds must be lower inside the house, since some of the ions would be deposited on the walls where entering the houses [3], [25]. There could also be other explanations for an interaction between power lines and domestic radon in relation to childhood leukemia; for instance if the exposure to power lines makes the child more vulnerable to exposure to radon, or vice versa.

The interpretation of the observed interaction is not straightforward since it is not only based on a statistically significant higher risk among those exposed to high radon and living close to a power line but also a statistically significant lower risk among those being exposed to low domestic radon and living 200–599 meters from a power line, which is not easy to explain. The observed interaction might be due to chance because of few cases and controls in several cells. The finding of no statistically significant interaction when the 75^th^ percentile was used as cut-off also suggests that the interaction found when using the median as cut-off might be due to chance. Even if the interaction between power lines and radon in relation to childhood leukemia is true, the potential effect in terms of absolute risk would be very small.

We found almost no change in the estimated association between distance to power lines and risk of childhood leukemia when adjusting for socioeconomic status of the municipality, urbanization, maternal age, birth order, domestic radon and traffic-related air pollution. This is in line with other studies investigating the association between ELF-MF and childhood leukemia, where the inclusion of potential confounders had no substantial effect on the estimates [5]–[18].

## Conclusion

The statistically significant interaction observed between power lines and radon might be due to chance since numbers were small and the interaction was no longer significant when changing the cut-point for radon exposure. We found no support for an interaction between power lines and traffic-related air pollution and no change in the estimated association between distance to power lines and the risk of childhood leukemia by adjustment for potential confounding factors.

## Supporting Information

Figure S1 **Number of cases and controls in the study.**
(TIF)

Table S1 **The joint effects of distance to nearest power line and domestic radon and air pollution, respectively, on leukemia.**
(DOCX)

Table S2 **The joint effects of distance to nearest power line and domestic radon and air pollution, respectively, on leukemia.**
(DOCX)

Table S3 **The joint effects of distance to nearest power line and domestic radon on leukemia for the period 1968–1994.**
(DOCX)

Table S4 **The joint effects of distance to nearest power line and domestic radon on leukemia risk with exact methods and with asymptotic methods.**
(DOCX)
