# Supplementary figures and images for: Distance to High-Voltage Power Lines and Risk of Childhood Leukemia – an Analysis of Confounding by and Interaction with Other Potential Risk Factors
Source: PLoS One. 2014 Sep 26;9(9):e107096. doi: 10.1371/journal.pone.0107096 (PMC4178021; doi:10.1371/journal.pone.0107096)

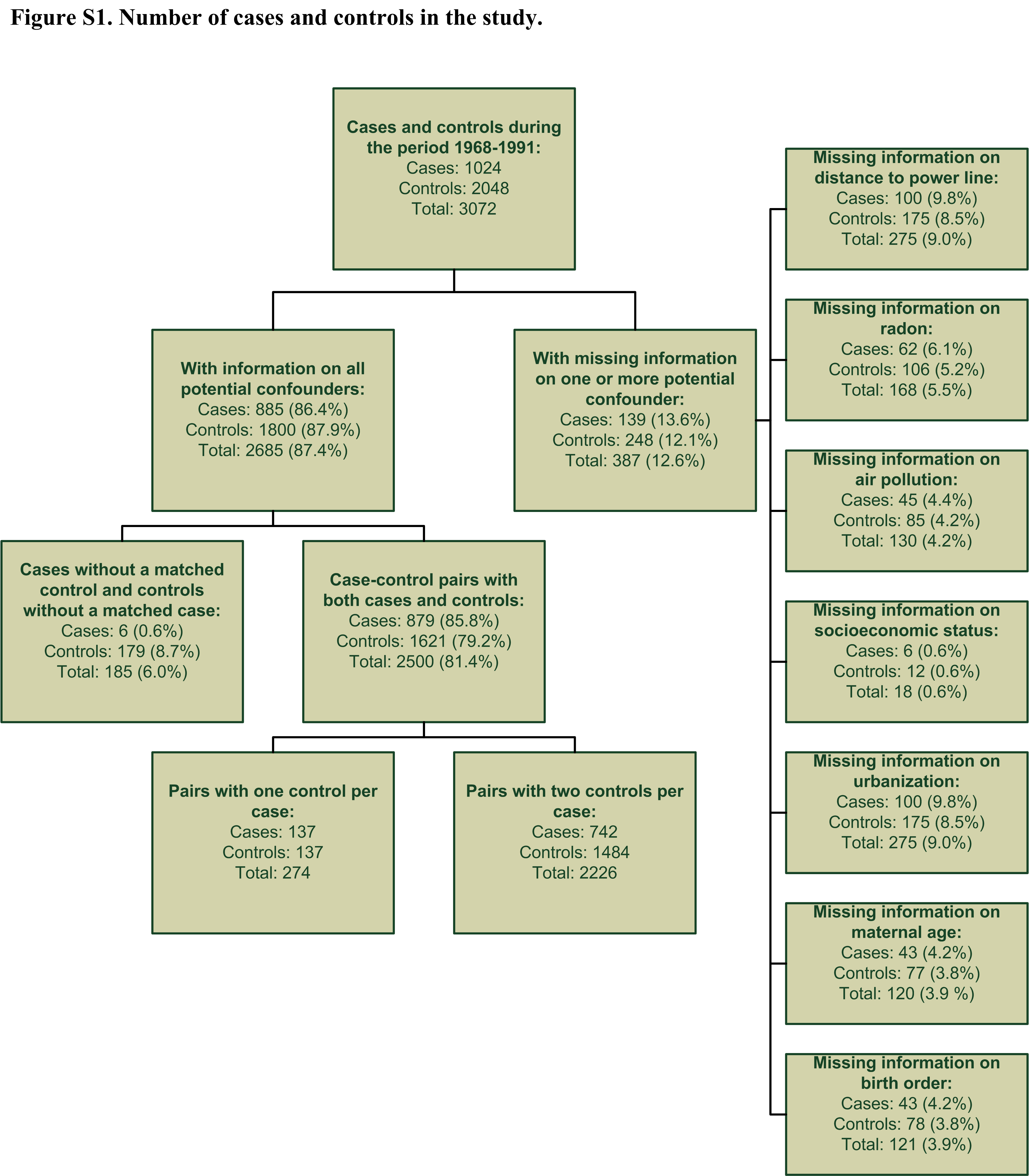

Supplement: Figure S1 — Number of cases and controls in the study. (TIF) [file pone.0107096.s001.tif]
