# Supplementary material for: Distance to High-Voltage Power Lines and Risk of Childhood Leukemia – an Analysis of Confounding by and Interaction with Other Potential Risk Factors
Source: PLoS One. 2014 Sep 26;9(9):e107096. doi: 10.1371/journal.pone.0107096 (PMC4178021; doi:10.1371/journal.pone.0107096)
Supplement: Table S1 — The joint effects of distance to nearest power line and domestic radon and air pollution, respectively, on leukemia. (DOCX) [file pone.0107096.s002.docx]

**Table S1. The joint effects of distance to nearest power line and domestic radon and air pollution, respectively, on leukemia.**

|  | Adjusted | | | |
| --- | --- | --- | --- | --- |
|  | RR (95% CI)  (N cases; N controls) | | | P-value for interaction |
|  | Distance (meters) | | |  |
|  | 0-199 | 200-599 | ≥600 |  |
| Domestic radon (Bq/m^3^)^1, 2^ |  |  |  | 0.90 |
| <75 | 1.54 (0.48-4.90) | 0.63 (0.34-1.15) | 1.00 |  |
|  | (5; 7) | (15; 45) | (626; 1177) |  |
| ≥75 | 1.99 (0.57-6.95) | 0.83 (0.44-1.58) | 1.09 (0.89-1.33) |  |
|  | (5; 5) | (14; 32) | (214; 355) |  |
| NO_x_ at the front door (ppb)^1, 3^ |  |  |  | 0.59 |
| <13 | 1.91 (0.75-4.86) | 0.72 (0.46-1.14) | 1.00 |  |
|  | (9; 9) | (28; 69) | (646; 1112) |  |
| ≥13 | 0.71 (0.07-6.91) | 0.26 (0.03-2.29) | 0.83 (0.67-1.03) |  |
|  | (1; 3) | (1; 8) | (194; 420) |  |

^1^ Cut-point is the 75^th^ percentile

^2^ The adjusted analysis includes following potential confounders: socioeconomic status, urbanization, maternal age, birth order and air pollution

^3^ The adjusted analysis includes following potential confounders: socioeconomic status, urbanization, maternal age, birth order and domestic radon
