# Supplementary material for: Distance to High-Voltage Power Lines and Risk of Childhood Leukemia – an Analysis of Confounding by and Interaction with Other Potential Risk Factors
Source: PLoS One. 2014 Sep 26;9(9):e107096. doi: 10.1371/journal.pone.0107096 (PMC4178021; doi:10.1371/journal.pone.0107096)
Supplement: Table S2 — The joint effects of distance to nearest power line and domestic radon and air pollution, respectively, on leukemia. (DOCX) [file pone.0107096.s003.docx]

**Table S2. The joint effects of distance to nearest power line and domestic radon and air pollution, respectively, on leukemia.**

|  | Adjusted | | | |
| --- | --- | --- | --- | --- |
|  | RR (95% CI)  (N cases; N controls) | | | P-value for interaction |
|  | Distance (meters) | | |  |
|  | 0-199 | 200-599 | ≥600 |  |
| Domestic radon (Bq/m^3^)^1, 2^ |  |  |  |  |
| <19 | 0.56 (0.06-5.13) | 0.16 (0.02-1.29) | 1.00 | 0.31 |
|  | (1; 4) | (1; 12) | (213; 424) |  |
| 19-63 | 2.91 (0.48-17.83) | 0.68 (0.30-1.54) | 1.05 (0.84-1.33) |  |
|  | (3; 2) | (8; 24) | (352; 623) |  |
| >63 | 2.07 (0.65-6.57) | 0.89 (0.50-1.58) | 1.04 (0.81-1.34) |  |
|  | (6; 6) | (20; 41) | (275; 485) |  |
| NO_x_ at the front door (ppb)^1, 3^ |  |  |  |  |
| <7 | 2.44 (0.73-8.14) | 0.54 (0.25-1.16) | 1.00 | 0.55 |
|  | (6; 5) | (9; 33) | (281; 497) |  |
| 7-11 | 3.03 (0.50-18.36) | 1.08 (0.55-2.11) | 1.18 (0.95-1.48) |  |
|  | (3; 2) | (15; 26) | (305; 484) |  |
| >11 | 0.48 (0.05-4.22) | 0.57 (0.20-1.59) | 0.90 (0.70-1.14) |  |
|  | (1; 5) | (5;18) | (254; 551) |  |

^1^ Cut-point for radon and air pollution is tertiles

^2^ The adjusted analysis includes following potential confounders: socioeconomic status, urbanization, maternal age, birth order and air pollution

^3^ The adjusted analysis includes following potential confounders: socioeconomic status, urbanization, maternal age, birth order and domestic radon
