# Supplementary material for: Distance to High-Voltage Power Lines and Risk of Childhood Leukemia – an Analysis of Confounding by and Interaction with Other Potential Risk Factors
Source: PLoS One. 2014 Sep 26;9(9):e107096. doi: 10.1371/journal.pone.0107096 (PMC4178021; doi:10.1371/journal.pone.0107096)
Supplement: Table S3 — The joint effects of distance to nearest power line and domestic radon on leukemia for the period 1968–1994. (DOCX) [file pone.0107096.s004.docx]

**Table S3. The joint effects of distance to nearest power line and domestic radon on leukemia for the period 1968-1994.**

|  | Not adjusted | | | |
| --- | --- | --- | --- | --- |
|  | RR (95% CI)  (N cases; N controls) | | | P-value for interaction |
|  | Distance (meters) | | |  |
|  | 0-199 | 200-599 | ≥600 |  |
| Domestic radon (Bq/m^3^)^1^ |  |  |  |  |
| <42 | 0.22 (0.03-1.71) | 0.33 (0.12-0.87) | 1.00 | 0.01 |
|  | (1; 9) | (5; 28) | (503; 932) |  |
| ≥42 | 2.62 (0.97-7.07) | 0.96 (0.61-1.51) | 1.03 (0.88-1.20) |  |
|  | (9; 7) | (31; 59) | (485; 886) |  |

^1^ Cut-point is the median
