# Supplementary material for: Distance to High-Voltage Power Lines and Risk of Childhood Leukemia – an Analysis of Confounding by and Interaction with Other Potential Risk Factors
Source: PLoS One. 2014 Sep 26;9(9):e107096. doi: 10.1371/journal.pone.0107096 (PMC4178021; doi:10.1371/journal.pone.0107096)
Supplement: Table S4 — The joint effects of distance to nearest power line and domestic radon on leukemia risk with exact methods and with asymptotic methods. (DOCX) [file pone.0107096.s005.docx]

**Table S4. The joint effects of distance to nearest power line and domestic radon on leukemia risk with exact methods and with asymptotic methods.**

|  | Exact test (unadjusted) | | | Asymptotic test (unadjusted) | | |
| --- | --- | --- | --- | --- | --- | --- |
|  | RR (95% CI)  (N cases; N controls) | | | RR (95% CI)  (N cases; N controls) | | |
|  | Distance (meters) | | | Distance (meters) | | |
|  | 0-199 | 200-599 | ≥600 | 0-199 | 200-599 | ≥600 |
| Domestic radon (Bq/m^3^)^1^ |  |  |  |  |  |  |
| <42 | 0.31 (0.04-2.63) | 0.24 (0.07-0.82) | 1.00 | 0.31 (0.01-2.62) | 0.24 (0.05-0.82) | 1.00 |
|  |  |  |  |  |  |  |
| ≥42 | 3.06 (1.08-8.66) | 0.86 (0.53-1.39) | 1.03 (0.87-1.23) | 3.06 (0.97-10.5) | 0.86 (0.51-1.42) | 1.03 (0.87-1.23) |

^1^ Cut-point is the median
